# Potential Modulatory Role of Phoenixin-14 in Epithelial–Mesenchymal Transition of Endometriotic 12Z Cells

**DOI:** 10.3390/biomedicines13010158

**Published:** 2025-01-10

**Authors:** Karolina Iwona Kulinska, Magdalena Wierzbicka, Anna Dera-Szymanowska, Krzysztof Szymanowski, Mirosław Andrusiewicz, Maria Wołuń-Cholewa

**Affiliations:** 1 Department of Cell Biology, Poznan University of Medical Sciences, 60-806 Poznań, Poland; biotechwierzbicka@gmail.com (M.W.); andrus@ump.edu.pl (M.A.); doskon@ump.edu.pl (M.W.-C.); 2 Clinic of Perinatology and Gynaecology, Poznan University of Medical Sciences, 60-535 Poznań, Poland; annaszerszen@wp.pl (A.D.-S.); kp.szymanowski@wp.pl (K.S.)

**Keywords:** endometriosis, 12Z human ectopic epithelial cell line, phoenixin-14 (PNX-14), epithelial cadherin (E-cadherin/*CDH1*), thrombospondin 2 (*THBS2*)

## Abstract

**Background/Objectives**: Endometriosis is a painful chronic condition in which the endometrium grows outside the uterus. The epithelial–mesenchymal transition (EMT) is critical to endometriosis progression, where cells lose epithelial traits and gain invasiveness. **Methods**: This study investigates the effects of phoenixin-14 (PNX-14), a neuropeptide found at reduced levels in endometriosis patients, on the expression of two molecular EMT markers, CDH1 (E-cadherin) and THBS2 (thrombospondin 2), as well as cell viability in the endometriosis-derived 12Z cell line. Cells were treated with physiological (0.2 nM) and endometriosis-relevant (0.05 nM) concentrations of PNX-14. Gene expression was analyzed using RT-qPCR, while protein localization was assessed by immunocytochemistry. Cell viability was measured using an XTT assay. **Results**: *THBS2* gene expression was significantly decreased, and *CDH1* remained unchanged in cells stimulated by 0.05 nM PNX-14. Immunolocalization indicates a weaker THBS2 and CDH1 protein immunosignal reaction for 0.05 nM PNX-14. PNX-14 treatment also exhibited a biphasic effect on cell viability. Lower concentration initially decreased viability at 48 h but then significantly increased it at 72 h. This increase coincided with the decrease in *THBS2* expression, suggesting a potential link between PNX-14, *THBS2*, and cell viability. **Conclusions**: A negative correlation between cell viability and the expression of both EMT markers further highlights their possible involvement in the survival and adaptability of ectopic epithelial cells. Our findings suggest a complex interplay between PNX-14, EMT markers, and cell viability in ectopic epithelial cells. PNX-14’s ability to modulate these factors warrants further investigation to elucidate its role in endometriosis.

## 1. Introduction

Endometriosis is a chronic disease affecting 6–10% of reproductive-aged women. It is manifested by the presence of stroma and glandular epithelial cells of the endometrium outside the uterine cavity [1,2]. In its advanced forms, the lesions create cysts and nodules located in the ovaries. The disease is usually detected at an advanced stage due to the lack of proper diagnosis and non-invasive monitoring of its progression [1,3]. The formation of an endometrial lesion depends on the successful adhesion, survival, invasion, and proliferation of endometrial fibroblasts and epithelial cells in a space outside the uterine cavity. One of the most important biological processes during the formation of new foci of ovarian endometriosis is the epithelial–mesenchymal transition (EMT) [4,5,6]. The EMT mechanism is crucial in differentiating cells into particular cell types and organizing cells into tissues with specific functions. It is also critical during tissue regeneration and aids wound healing [7]. EMT disruption occurs in many diseases, including cystic fibrosis, asthma, systemic lupus erythematosus, systemic scleroderma, Sjögren’s syndrome, rheumatoid arthritis, cancer, and endometriosis [5,8,9,10,11,12].

A cell undergoing EMT loses markers associated with the epithelial phenotype—E-cadherin (CDH1), cytokeratin, and claudins—and simultaneously acquires a mesenchymal, invasive character with increased vimentin, fibronectin, and N-cadherin [5]. The cells lose connections between neighboring cells and apical–basal polarity, increasing mobility. These changes induce increased cell invasion and survival. The ectopic endometrium exhibits partial EMT, in which epithelial cells gain expression of mesenchymal markers (e.g., N-cadherin) but retain epithelial cell features (restored expression of E-cadherin, B-catenin, and claudins) and the ability to adhere to neighboring cells [5].

The key factors inducing EMT in endometriosis are probably hypoxia, transforming growth factor beta, and the action of signaling pathways related to regulating plasma estradiol levels found in women suffering from the disease [5,8,13]. Additionally, stromal fibroblasts in the ectopic microenvironment further enhance the transformation, stimulating epithelial proliferation while disrupting cellular polarity. This dynamic facilitates the formation of invasive structures that penetrate surrounding tissues, such as the mesothelium and ovarian parenchyma [5]. Understanding the molecular markers and physiological conditions driving these cellular transformations is critical for uncovering how this process contributes to disease progression [14].

One of the primary markers of EMT is the functional loss of CDH1, the protein involved in the cell–cell adhesion, mobility, and proliferation of epithelial cells [15]. In healthy endometrium, CDH1 is strongly expressed in epithelial glandular cells and weakly detected in the stroma [16]. In ectopic epithelial cells, the level of E-cadherin is reduced and related to the invasive phenotype of the cells [17]. Another protein strongly correlated with EMT markers is thrombospondin 2 (THBS2), a secretory protein frequently found in the extracellular matrix. It is localized mainly in endometrial glandular epithelium with *THBS2* gene expression from stromal fibroblasts [16].

The role of THBS2 is to regulate cell–cell and cell–extracellular matrix interactions and to activate transforming growth factor beta—a known stimulator of EMT [18]. THBS2 enhances the adhesion of platelets, melanoma cells, muscle cells, endothelial cells, fibroblasts, and epithelial cells and controls cytoskeletal dynamics, cell migration, and cell adhesion [19,20,21]. In endometrial cancer, an increased THBS2 is associated with increased angiogenesis and a worse prognosis [22]. The role of THBS2 in endometriosis is underinvestigated. Higher expression of this protein has been found in the ectopic endometrium of ovarian endometriosis compared to the eutopic endometrium in healthy women [4].

Phoenixin (PNX) is a newly discovered neuropeptide produced in the hypothalamus that regulates memory processes and functions of the reproductive system [23,24]. It occurs in two main isoforms, PNX-14 and PNX-20, which are formed from the precursor protein, small integral membrane protein 20. Regarding biological activity, the listed isoforms do not differ among themselves. These neuropeptides, via G protein-coupled receptor 173 (GPR173), activate the cAMP/PKA pathway, leading to the phosphorylation of the cAMP response element-binding protein [25].

PNX-14 regulates the hypothalamic–pituitary–gonadal axis by stimulating gonadoliberin receptor expression and indirectly increasing luteinizing hormone levels.

PNX is a neuropeptide that, among other processes, regulates reproductive system function, including ovarian follicle maturation and hypothalamic–pituitary–gonadal axis stimulation [23,26,27]. Women with endometriosis have significantly lower levels of serum PNX-14 and reduced expression of its receptor, GPR173, in ectopic ovarian lesions (lack of GPR173 protein within the ectopic glandular epithelium) [28]. PNX-14 and its receptor GPR173 are expressed predominantly in fibroblasts of the endometrial stroma but are also found in ectopic and eutopic epithelial cells [28]. PNX-14 increases the migration and proliferation of 12Z endometriosis epithelial cells, but the effect differs between physiological and endometriosis-related neuropeptide concentrations.

PNX-14 increased the migration of endometriosis epithelial cells (12Z) pre-incubated with a concentration of PNX-14 characteristic of women with endometriosis, toward the concentration observed in healthy women, which enhanced epithelial cell proliferation [29]. This observation indicated the potential role of PNX-14 in the biology of ectopic epithelial cells. It led us to investigate the effect of PNX-14 on the gene expression levels of proteins engaged in migration and the EMT process. It is unknown whether PNX affects EMT by altering the expression of transition markers. This study aimed to determine the impact of PNX-14 at the concentrations observed in diseased and healthy women on the expression of *CDH1* and *THBS2* and the viability of the 12Z ectopic epithelial cell line.

## 2. Materials and Methods

### 2.1. Cell Line Culture

The 12Z cell line is a well-established and widely used model that closely mimics the characteristics of endometriotic lesions. Derived from human endometriotic tissue, 12Z cells exhibit key features of the disease, such as invasiveness, angiogenic potential, and resistance to apoptosis. Additionally, 12Z cells respond predictably to hormonal and inflammatory stimuli, enabling the exploration of disease pathways and potential therapeutic targets. Their reproducibility and versatility in experimental applications, such as molecular assays and drug testing, further support their use as a reliable and relevant model in endometriosis research [30,31,32]. The 12Z cell line (Applied Biological Materials Inc., Richmond, Canada) was cultured under standard conditions (5% CO_2_, 37 °C) in HAM’s F12 medium supplemented with 10% fetal bovine serum (FBS), 1.8 g/L D-glucose, 110 mg/L sodium pyruvate, 2 mM L-glutamate, 100 U/mL penicillin, and 100 μg/mL streptomycin (Biowest, Nuaillé, France). After five days, the cells reached 90% confluence and were harvested, counted, and used for the next steps of the study.

### 2.2. Stimulation with PNX-14

For immunocytochemistry analyses, the 12Z cells were cultured in tissue culture standard dishes ø 9 cm (5 × 10^5^ cells/per dish) with immersed sterile coverslips. A 6-well plate (1 × 10^5^ cells per well) was used for mRNA expression analyses, and XTT cellular viability and cytotoxicity assays were performed in 96-well plates (5 × 10^3^ cells per well). After 24 h, cells were washed with phosphate-buffered saline buffer (PBS; Sigma Aldrich, Darmstadt, Germany), and then 0.05 nM or 0.2 nM PNX-14 (Novazym, Poznan, Poland) dissolved in F12 medium was added to reach a final volume of 10 mL (ø 9 cm dishes), 2 mL (6-well plates), or 100 µL (96-well plates). The 0.05 nM concentration was calculated based on the median concentration of PNX-14 found in the serum of 32 women with ovarian endometriosis (Me = 76.7 pg/mL, [Q1–Q3] = [51.1–101.6]) [28], and 0.2 nM was established as the median of PNX-14 concentrations found in the serum of 23 healthy controls (Me = 324.8 pg/mL, [Q1–Q3] = [214.9–399.3]) [28]. The PBS buffer served as a control. Incubation lasted 72 h. The medium was changed every 24 h with freshly prepared concentrations of PNX-14.

In the case of immunocytochemistry per each concentration of PNX-14 and each protein localization and control, six coverslips were analyzed in duplicates (2 × antibodies’ specificity controls, 6 × PBS controls, and for THBS2 and CDH1 separately: 6 × PNX-14 [0.2 nM], 6 × PNX-14 [0.05 nM]). Quantitative expression analysis was performed for six independent replications in duplicates (6 wells × PBS controls, 6 wells × PNX-14 [0.2 nM], and 6 wells × PNX-14 [0.05 nM]). For XTT, two independent cultured plates were used with 24 wells × PBS controls, 24 wells × PNX-14 [0.2 nM], and 24 wells × PNX-14 [0.05 nM] in duplicates.

### 2.3. CDH1 and THBS2 Expression Analysis

#### 2.3.1. RNA Quality and Quantity Assessment

Cells grown on 6-well plates after 72 h of incubation with PNX-14 were lysed with TriReagent solution (Bioshop, Burlington, ON, Canada) and stored at −80 °C until the RNA isolation step according to the phenol-chloroform method [33]. For each PNX-14 concentration, the experiments were conducted in 8 independent experiments.

The RNA concentration was measured on a UV spectrophotometer, NanoPhotometer^®^ NP-80 (IMPLEN, München, Germany). The quality and integrity of the RNA were evaluated using 1% agarose gel electrophoresis in a denaturing FA buffer (200 mmol/L 3-(N-morpholino) propanesulfonic acid, 50 mmol/L sodium acetate, 10 mmol/L ethylenediaminetetraacetic acid, 246 mmol/L formaldehyde; LabEmpire For Science, Rzeszów, Poland). Samples with visible 18S and 28S ribosomal RNA bands were used for reverse transcription, followed by quantitative polymerase chain reactions (RT-qPCR).

#### 2.3.2. Reverse Transcription

RNA at the final concentration of 50 ng/μL was used for reverse transcription reactions using a Transcriptor First Strand cDNA Synthesis Kit (F. Hoffmann-La Roche Ltd., Basel, Switzerland), *Escherichia coli* poly(A) polymerase, and dATP (Carolina Biosystems, Prague, Czech Republic).

In the first step, the mixture of the RNA, water, and primers (5 pmol/mL of oligo (d)T_10_ and 1 pmol/mL of random hexamer primer) were denatured at 65 °C for 10 min and subsequently chilled on ice. Next, enzymes, deoxynucleotide triphosphates (dNTPs), and RNase inhibitors were added to each sample as follows: 0.5 U/mL Transcriptor Reverse Transcriptase, 0.25 U/mL RNase Inhibitor, 1× Reverse Transcriptase Buffer, 1 mmol/L of each dNTP (Roche), 0.1 U/mL *E. coli* poly(A) polymerase, and 0.1 mmol/L dATP (Carolina Biosystems). Reverse transcription was processed at 25 °C for 10 min, 55 °C for 30 min, and 85 °C for 5 min. After these reactions, the samples were cooled to 4 °C and stored at −20 °C until the qPCR reactions were performed (but not longer than one week).

#### 2.3.3. Quantitative Polymerase Chain Reaction

Based on previous studies, beta-2-microglobulin (*B2M*) is one of the most stable genes in ectopic endometrium studies and was chosen as a reference gene [34]. The level of *B2M* expression was assessed using LightCycler^®^ 2.0 (Roche Diagnostic GmbH, Basel, Switzerland) and Eva Green as a fluorescent dye (Solis BioDyne, Tartu, Estonia). The reaction mixture comprised 1× HotFire Pol EvaGreen qPCR Mix Plus (Solis BioDyne) and 5 pmol/μL forward and reverse high-performance liquid chromatography purified primers (Genomed, Gdańsk, Poland; Table 1). qPCR was conducted in the following steps: preincubation at 95 °C for 12 min, then 45 cycles with endpoint acquisition: 95 °C for 15 s, 60 °C for 30 s, and 72 °C for 20 s. The melting curve was analyzed at 95 °C for 0 s, 65 °C for 15 s, and 97 °C (with a ramp rate of 0.1 °C/s). For each sample set, the reactions were performed in duplicates.

The expression of *CDH1* and *THBS2* was analyzed using specific primers and TaqMan hydrolysis 8-mer locked nucleic acid probes with fluorescein (FAM) modified 5′ end and black hole quencher (BHQ-2) at the 3′ end (#77, cat. no. 0466689003001; Roche Diagnostic GmbH, Basel, Switzerland). Gene-specific primers and probes were designed using the Universal Probe Library Assay Design Center (Roche Applied Science, discontinued). The reaction mixture was composed of 1× LightCycler^®^ TaqMan^®^ master mix (Roche Diagnostic GmbH), 200 nM TaqMan probes, and 5 pmol/uL of both primers (respectively for *CDH1* or *THBS2* reactions; Table 1). The thermal profile for each reaction was as follows: a preincubation step at 95 °C for 12 min, then 45 cycles: 95 °C for 15 s, 60 °C (annealing/extension) for 30 s, and fluorescence acquisition at 72 °C for 1 s.

Each reaction was conducted in duplicates using a LightCycler^®^ 2.0 (Roche Diagnostic GmbH), and the gene expression level was normalized to the expression of reference *B2M* [28,34]. For each gene, the amplification efficiency was determined using standard curves. The concentration ratio (Cr) was used to compare the relative expression of analyzed genes between untreated and PNX-14-stimulated 12Z cells (of both concentrations).

### 2.4. Immunocytochemistry

After 72 h of incubation with PNX-14, 12Z cells (cultured on the Petri dishes with immersed coverslips) were fixed in 4% paraformaldehyde (Chempur; Piekary Śląskie, Poland) for 20 min and then washed and stored in PBS until further treatment. Immediately before the staining procedure, cells were washed with 5 mL PBS buffer (3 × 5 min) and immersed in 5 mL of Tris/EDTA buffer (pH 9; Agilent Technologies, Santa Clara, CA, USA). Antigens were retrieved by microwave heating (600W for 10 min) in a water bath. Subsequently, cells were cooled at room temperature, rinsed with PBS buffer (3 × 5 min), placed on microscope slides in a humidity chamber, and the immunocytolocalization of CHD1 and THBS2 was carried out.

#### 2.4.1. Cadherin 1 Immunolocalization

The slides with cells prepared as described above were incubated for one hour in blocking buffer—PBS-T (PBS, 0.1% Tween 20, Sigma Aldrich, Darmstadt, Germany) supplemented with 3% bovine serum albumin fraction V—and then incubated in the darkness with ready-to-use primary polyclonal rabbit anti-CDH1 antibodies (cat. PA1-37203; Thermo Scientific, Waltham, MA, USA) at 4 °C overnight. Next, slides were washed with PBS-T buffer (3 × 5 min) and subsequently incubated with 1:500 PBS-T diluted horse anti-rabbit secondary antibodies conjugated with fluorescent DyLight 488 dye (cat. DI-1088-1.5; Vector Laboratories, Inc., Newark, CA, USA) at room temperature in the darkness for 1 h. After washing, cells were incubated with 1 µg/mL of 4′,6-diamidino-2-phenylindole (DAPI) solution in PBS at room temperature in the darkness for 5 min (ThermoFisher Scientific, Carlsbad, CA, USA) for counterstaining. The slides were sealed with a fluorescence mounting medium (Agilent Technologies, Santa Clara, CA, USA) and observed under the LSM 780 confocal microscope (Carl Zeiss Meditec AG, Jena, Germany). In the negative control reactions, the blocking buffer solution replaced the primary antibodies to check the antibodies’ specificity.

#### 2.4.2. Thrombospondin 2 Immunolocalization

For THBS2 protein localization, cells were incubated in a 3% hydrogen peroxide solution for 3 min to block endogenous peroxide activity, and then unspecific epitope binding was blocked with goat milk for 1 h. After the blocking step, 12Z cells were incubated overnight at 4 °C in PBS-T buffer with a 1:500 diluted goat polyclonal THBS2 antibody (cat. sc-12313; Santa-Cruz, TX, USA). The next day, slides were washed in PBS-T buffer (3 × 10 min) and incubated with a 1:200 donkey anti-goat secondary antibody conjugated with horseradish peroxide (cat. 061005; Bio-Rad AbD Serotec GmbHSerotec, Neuried, Germany) at room temperature in the darkness for 1 h. After that, cells were rinsed and incubated with 3,3′-diaminobenzidine tetrahydrochloride substrate solution (DAB; Agilent Technologies, Santa Clara, CA, USA) to trigger the enzyme–dye reaction. The reaction was stopped in the water, and the slides were sealed with Dako Ultramount Aqueous Permanent Mounting Medium (Agilent Technologies, Santa Clara, CA, USA) and viewed under an Axiophot light microscope (Carl Zeiss Meditec AG, Göttingen, Germany). The blocking buffer solution replaced the primary antibodies in the negative control reactions.

### 2.5. XTT Viability and Cytotoxicity Assays

After 24 h, 12Z cells were seeded onto 96-well plates, and 100 µL PNX-14 in F12 medium in the final concentrations of 0.05 nM and 0.2 nM (dissolved in HAM’s medium) was added into each well and incubated for 24, 48, and 72 h (PBS was used as a control). The medium was changed every 24 h (with the same concentrations of PNX-14).

XTT sodium salt powder (2,3-bis(2-methoxy-4-nitro-5-sulfophenyl)-2H-tetrazolium -5-carboxanilide; cat. XTT101.500, Bioshop, Canada) was prepared in HAM’s F12 medium without FBS at the stock concentration of 1 mg/mL (3 mM) and sterilized using a syringe filter of 0.2 µm. Menadion (2-methyl-1,4-naphthoquinone; cat. M5625-25G, Sigma Aldrich, Darmstadt, Germany) was prepared as a 10 mM solution in absolute alcohol (cat. PBA6480111-0500, Alfachem, Lublin, Poland). Next, XTT solution, F12 medium with FBS, and menadion were mixed to prepare a ready-to-use XTT reagent with a final concentration of 1.5 mM with 100 µM of menadion.

One hundred microliters of the prepared XTT solution was added to the cells and incubated at 37 °C for 4 h. Cells were examined spectrophotometrically at the test wavelength of 450 nm and the 692 nm reference using an EPOCH-S1 microplate reader (Agilent Technologies, Santa Clara, CA, USA).

### 2.6. Statistical Analysis

The normality of the obtained distributions was evaluated using the Shapiro–Wilk test. A multivariate analysis of variance (ANOVA) for repeated measurements was performed, and if the sphericity assumption was not met, epsilon correction and multivariate analysis of variance (MANOVA) with Tukey’s honest significance post hoc test were applied. The Pearson correlation test was used to evaluate the strength of the correlation coefficient (r). The strength of the correlation coefficient was assessed according to Guilford’s classification. When the *p*-value was higher than 0.05, the results were considered non-significant. Statistical calculations were performed using Statistica (version 13; TIBCO Software, Palo Alto, CA, USA & StatSoft, Kraków, Poland) and PQStat Software (version: v.1.8.0.476; PQStat, Poznań, Poland). The visualizations were achieved using JMP software (version: 17.2.0 (701896), JMP Statistical Discovery LCC, Cary, NC, USA).

## 3. Results

### 3.1. CDH1 and THBS2 Expression

**Figure 1:**
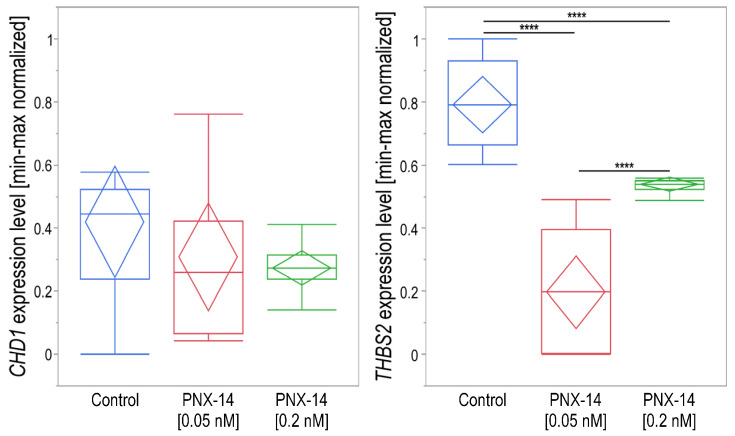
Relative expression analysis of *CDH1* and *THBS2* (normalized to *B2M* and after min-max rescaling) for 12Z cells unstimulated and stimulated with 0.05 and 0.2 nM PNX-14 for 72 h. Data are presented as median [interquartile range], min-max values, and confidence diamond in the middle. **** *p* < 0.001; PNX-14—phoenixin-14, *CDH1*—cadherin 1, *THBS2*—thrombospondin 2.

The mean threshold cycles (the qPCR cycle in the fluorescence crossed the background border) for *CDH1* was much higher (mean ± standard deviation, 30.59 ± 1.21) than *THBS2* (20.76 ± 1.68), which means that the relative expression of *CDH1* was at a very low level. No significant differences were observed in *CDH1* expression after 72 h in cells stimulated with different concentrations of PNX-14 compared to controls (*p* = 0.1710). Statistical analysis showed that approximately 34% of the intrasubject variability in the results could be explained by PNX-14 stimulation (η^2^ = 0.34). For *THBS2*, significant differences were found between the control and tested concentrations (*p* < 0.0001). The highest level of *THBS2* was significantly observed in control cells compared to the lowest expression in cells stimulated with 0.05 nM PNX-14 (*p* = 0.0001) and intermediate level at the physiological concentration of 0.2 nM PNX-14 (*p* = 0.0007). Both concentrations of PNX-14 contributed to a significant difference in *THBS2* expression levels (*p* = 0.0002; Figure 1). Over 87% of the intrasubject variability in gene activity results could be explained by PNX-14 cell stimulation (η^2^ = 0.87).

### 3.2. Immunocytochemical Localization of CDH1 and THBS2 in 12Z Cells

**Figure 2:**
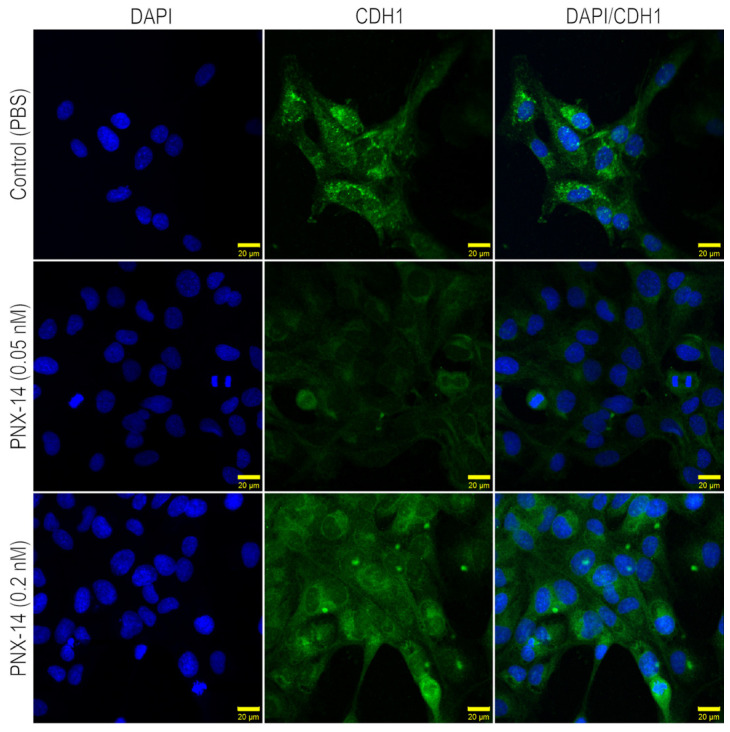
Assessment of CDH1 localization in 12Z epithelial cells unstimulated and stimulated with 0.05 and 0.2 nM PNX-14 for 72 h. CDH1 is localized as granules in the cytoplasm (green). A distinct pattern of protein aggregation and localization compared to the control was observed in cells stimulated with 0.05 nM PNX-14. The PNX-14 in the concentration of 0.2 nM showed a similar pattern of CHD1 as unstimulated control. The nuclei were co-stained with DAPI dye (blue). PNX-14—phoenixin-14, CDH1—cadherin 1, PBS—phosphate-buffered saline, DAPI—4′,6-diamidino-2-phenylindole.

**Figure 3:**
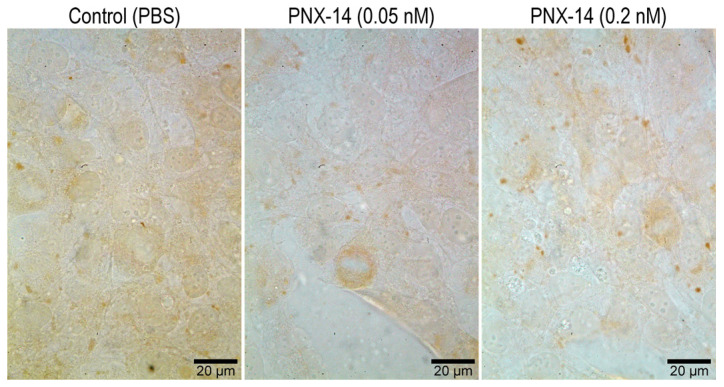
Assessment of THBS2 localization in 12Z epithelial cells unstimulated and stimulated with 0.05 and 0.2 nM PNX-14 for 72 h. Cytoplasmic localization. The strongest immunocytochemical reaction was observed in the control, then 0.2 nM PNX-14, and the weakest signal was detected in cells incubated with 0.05 nM PNX-14. PNX-14—phoenixin-14, PBS—phosphate-buffered saline.

Immunocytochemical analysis of 12Z cells, both unstimulated and stimulated with PNX-14 at concentrations of 0.05 nM and 0.2 nM, revealed the presence of CDH1 and THBS2 at the protein level. Representative images obtained using confocal (CDH1) and bright-field (THBS2) microscopy are shown in Figure 2 and Figure 3, respectively.

Depending on the concentration of PNX-14, a different pattern of CDH1 localization is visible. In unstimulated cells, CDH1 gives the strongest fluorescent signal, and the protein is mainly localized in the cytosol and the perinuclear region. Upon stimulation with 0.05 nM of PNX-14, the fluorescence of CDH1 decreased and was only visible in individual cells. A fourfold higher concentration of PNX-14 induces a higher fluorescence intensity of CDH1 than 0.05 nM but lower than in the control group (Figure 2).

Slight differences were observed in THBS2 among the various groups regarding immunocytochemical reaction strength (which was very weak; Figure 3). The 12Z control cells and those stimulated with PNX-14—at a 0.2 nM concentration—showed stronger immunofluorescence reaction signals than those stimulated with PNX-14 at 0.05 nM concentration.

### 3.3. XTT Viability and Cytotoxicity Assay

**Figure 4:**
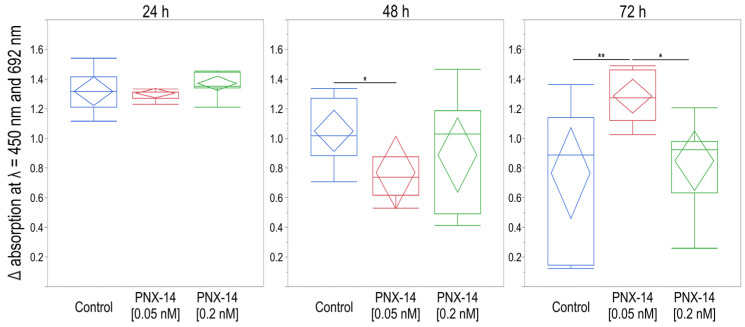
Viability analysis of 12Z cells unstimulated and stimulated with 0.05 nM and 0.2 nM PNX-14 for 24, 48, and 72 h. Results represent the mean differences (Δ) in absorbance measured at wavelengths λ = 450 nm and λ = 692 nm. Data are presented as median [interquartile range] and min-max values with a confidence diamond in the middle. * *p* < 0.05, ** *p* < 0.01. PNX-14—phoenixin-14.

The results of the viability analysis of 12Z cells stimulated for 24 h with 0.05 nM and 0.2 nM of PNX-14 indicated no significant differences between the mean absorbance values (*p* = 0.1984; Figure 4). Statistical analysis showed that only just under 15% of the intrasubject variability in results could be explained by 24 h PNX-14 stimulation (η^2^ = 0.15). Viability analysis of 12Z cell line cells subjected to 48 h stimulation with 0.05 nM and 0.2 nM PNX-14 indicated significant differences in absorbance (*p* = 0.0432). Over 27% of intrasubject variability in cell viability results could be explained by PNX-14 cell stimulation (η^2^ = 0.27). Cell viability was significantly decreased in cells stimulated with 0.05 nM PNX-14 compared to the control group (*p* = 0.0349). The post hoc test revealed no significant differences in the viability results. Viability analysis of 12Z cells subjected to 72 h of PNX-14 stimulation indicated significant differences between the mean values of absorbance (*p* = 0.0048). Over 41% of intrasubject variability in the cell viability results could be explained by PNX-14 cell stimulation (η^2^ = 0.41). Significantly increased cell viability stimulated with 0.05 nM PNX-14 was observed compared to the control group (*p* = 0.0063) and the group stimulated with 0.2 nM PNX-14 (*p* = 0.0213). The viability of control cells and cells stimulated with 0.2 nM of PNX-14 was similar (*p* = 0.8451).

**Figure 5:**
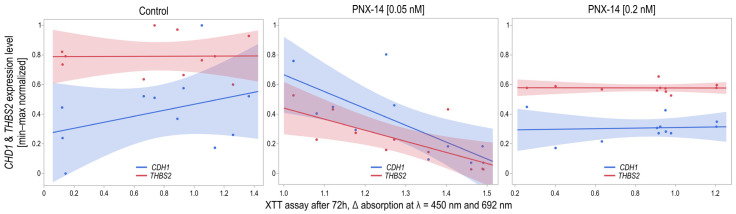
Correlation of XTT results with *CHD1* and *THBS2* expression in 12Z cells stimulated with 0.05 nM PNX-14 for 72 h. Spearman’s rank correlation dot-plot of *CHD1* and *THBS2* (normalized to *B2M* and after min-max rescaling) with XTT assay results. PNX-14—phoenixin-14, CDH1—cadherin 1, THBS2—thrombospondin 2, XTT—2,3-bis(2-methoxy-4-nitro-5-sulfophenyl)-2H-tetrazolium-5-carboxanilide.

A significant correlation was found between the results of the XTT test and the expression of both *CDH1* (*p* = 0.0097) and *THBS2* (*p* = 0.0122) when cells were incubated with 0.05 nM of PNX-14 for 72 h. For both genes, the correlation was negative and strong (*r* = −0.74 and *r* = −0.72). The variability in *CDH1* expression levels explains 54% of the variability in XTT test results, while for *THBS2*, the coefficient of determination was 52% (Figure 5). No significant correlations were found between the results of the XTT test and gene expression in the control sample and the physiological concentration of 0.2 nM PNX-14 (*p* > 0.05).

## 4. Discussion

Herein, we investigated the effects of PNX-14 on the metabolism and expression of cell-to-cell adhesion proteins in ectopic epithelial cells exposed to both physiological and endometriosis-specific concentrations of PNX-14 after 72 h of incubation time. We have observed, for the first time, reduced *THBS* expression and CDH1 protein level with the increased metabolic rate of endometriotic epithelial cells under endometriosis-specific PNX-14 stimulation.

### 4.1. PNX-14-Stimulated CDH1 and THBS2 Expression

We did not observe any changes in *CDH1* expression under physiological and endometriosis-specific PNX-14 stimulation at the RNA level. The complicated regulation of *CDH1* expression may explain the obtained results. Previous studies have shown a negative correlation of miR-542-3p with *CDH1* and *SMAD4* [35]. *CDH1* expression is regulated through different signaling pathways (e.g., Twist-1, Snail, Slug-1, ZEB1/2, and microRNA) to restore *CDH1* mRNA level, which may also interact with PNX-14 [36,37].

At the protein level, we observed a strong decrease in the accumulation of CDH1 under stimulation with endometriosis-specific concentrations of PNX-14 (0.05 nM), with no changes in CDH1 under 0.02 nM PNX-14. This observation might be accorded by studies that indicate the induction of lysosomal degradation of CDH1 as an early step in EMT [38,39,40].

*THBS2* expression decreased under both physiological and endometriosis-specific concentrations of PNX-14. Interestingly, cells incubated with PNX-14 at concentrations found in the serum of healthy women showed higher *THBS2* expression than those exposed to pathological concentrations. We also observed a slight decrease in THBS2 accumulation in 12Z endometrial epithelial cells incubated with endometriosis-specific concentrations of PNX-14 compared to control and physiological PNX.

*THBS2* encodes a protein critical for cell adhesion and extracellular matrix interactions, inhibiting angiogenesis and promoting apoptosis [41,42]. THBS2 in many cancers is a double-edged sword, exerting oncogenic and anti-angiogenic effects [20,43]. THBS2 in colorectal cancer is a prognostic biomarker, and its high expression was correlated with low overall and disease-free survival [20]. Interestingly, in colorectal cancer, a strong positive correlation was observed between the expression of *THBS2* and genes encoding the EMT markers N-cadherin, vimentin, TWIST1, and SNAIL, while there was a negative correlation with *CDH1* [20]. These results indicate that THBS2 is involved in the stimulation and development of EMT in cancer cells. Our study observed an opposite effect: a weak positive correlation of *THBS2* and *CDH1* expression (statistically insignificant). The role of THBS2 in ectopic epithelial cells might differ from that of cancer cells; lower expression of *THBS2* under PNX-14 stimulation may cause increased cell viability, metabolism, and migration. These findings are consistent with the study conducted on trophoblast cell lines when silencing *THBS2* by miR-942-5p led to increased proliferation, invasion, and angiogenesis [42]. The mechanism by which PNX-14 downregulates *THBS2* needs further research; of particular importance is the investigation of the effect of PNX-14 on circular and microRNA, which regulates the expression of genes involved in EMT, such as *CDH1* and *THBS2*.

Particularly noteworthy is the study of reduced expression for the CD36 thrombospondin receptor in the peritoneal macrophages of women with endometriosis, reducing their phagocytosis ability. Reduced THBS2 expression in epithelial cells stimulated with endometriosis-related PNX-14 concentrations may be responsible for the disrupted THBS2-CD36 signaling axis and impaired phagocytosis of the ectopic epithelium by macrophages, which contributes to the maintenance and proliferation of the endometrium outside the uterus [44,45].

### 4.2. PNX-14 Effect on 12Z Cell Viability

A 48 h exposure to an endometriosis-specific PNX-14 concentration reduced cell viability, but this effect reversed after 72 h. The physiological concentration of PNX-14 did not change 12Z cell viability. Moreover, we observed a strong negative correlation between the viability of 12Z cells (XTT assay) and the expression of *CDH1* and *THBS2* in cells treated with PNX-14 at endometriosis-specific concentrations.

The observed decrease in the viability of 12Z epithelial cells under the influence of 0.05 nM PNX-14 can be explained by the cytotoxic effect of endometriosis-related PNX-14 concentration and the increased—after 72 h—mean by activation of adaptive mechanisms, including an increase in migratory capacity, which is also related to the observed negative correlation between *CDH1* and *THBS2* expression and XTT results. No differences in viability were observed for a concentration of 0.2 nM, which may be consistent with studies conducted on preadipocytes, where a low concentration of 1 nM PNX-14 did not cause changes in viability. In contrast, high concentrations of 10 and 100 nM PNX-14 had a stimulatory effect on viability after 48 h of incubation [46]. Conversely, a previous study revealed that an endometriosis-specific PNX-14 concentration decreased the expression of phoenixin’s receptor with no change to the proliferation of ectopic epithelial cells [29]. Taken together, decreased *THBS2* expression, accompanied by increased cell metabolism, might be one of the mechanisms through which endometriosis-specific concentration of PNX-14 in a GPR173-dependent manner modulates the viability and apoptosis of epithelial cells [41,42].

An increase in the metabolism of ectopic epithelial cells with a concomitant decreased expression of THBS2 on gene and protein levels and a reduced concentration of CDH1 might indicate the stimulation of the EMT process of ectopic epithelial cells at PNX-14 concentrations observed in the serum of women with endometriosis.

### 4.3. PNX-14 and EMT Process of Ectopic Endometrium—Future Perspectives

The precise mechanism of PNX-14 within the cells of the ectopic endometrium remains elusive, particularly in the context of the interaction of fibroblasts with epithelial cells. It seems that the action of PNX may be paracrine—PNX-14 secreted by endometrial fibroblasts, which may interact with epithelial cells, causes an increase in their proliferation [29]. The hypothesis assumes that PNX-14 at low, endometriosis-specific concentrations might stimulate the EMT transition and increase the invasiveness of the ectopic endometrium. Conversely, physiological concentrations of this neuropeptide could restore the normal biology of the endometrial glandular epithelium, inhibiting EMT and fibroblast invasiveness. To better understand the potential applications of the results of this study, we propose to investigate the therapeutic implications of the dual action of PNX-14 in the ectopic endometrium. If validated, the hypothesis that PNX-14 exerts concentration-dependent effects—stimulating EMT and invasiveness at low endometriosis-specific levels while inhibiting these processes at physiological levels—could inform new treatment strategies. Specifically, therapies could target PNX-14 levels to restore normal epithelial and fibroblast interactions, thereby eliminating a key driver of endometriosis progression. Further studies are necessary to validate this hypothesis.

On the other hand, a high tissue concentration of 17β-estradiol (E2) might be one mechanism inhibiting or regulating PNX-14 production within the ectopic endometrium. Endometriosis is well established as an estrogen-dependent disease, and high E2 levels are known to promote EMT [47,48,49]. Studies in women with polycystic ovary syndrome revealed a negative correlation between PNX-14 and estradiol serum concentrations [50]. However, whether a similar correlation occurs at the tissue level or if any tissue-specific mechanisms regulate PNX-14 secretion from ectopic endometrium remains unclear. Interestingly, gonadotropin-releasing hormone analogs, used among others in the treatment of endometriosis, increase small integral membrane protein 20 gene expression and decrease *GPR173* in the rats’ hypothalamus, pituitary, and ovaries [51]. Based on the literature data, we can speculate that the off-axis effects of PNX-14 might depend on both gonadotropin-releasing hormone and E2 [51]. These insights could facilitate the design of combination therapies that leverage both hormonal and peptide-modulating strategies to improve patient outcomes. Ultimately, understanding the mechanisms of PNX-14 in EMT and ectopic endometrium biology could pave the way for innovative treatments, targeting the root causes of endometriosis and improving the quality of life for affected individuals.

### 4.4. Study Limitations

This study uses the 12Z cell line, a simplified 2D model of endometriosis. While this model provides valuable insights, it does not fully replicate the complexity of the in vivo milieu of ectopic lesions. In particular, it lacks the complicated interactions between fibroblasts and epithelial cells, as well as the influence of the unique microenvironment on EMT [18]. The analysis focused on a limited subset of EMT-related genes, which may not capture the full spectrum of EMT activity in endometriosis. Expanding the gene panel to include additional markers may provide a more comprehensive understanding of EMT and uncover novel pathways or interactions. Although our study examined gene expression, there may be differences between gene expression and protein activity. Future research investigating protein levels of EMT markers would provide critical insights into the functional role of these markers in disease progression. In addition, the inclusion of markers previously identified in the peripheral blood of endometriosis patients—such as SNAIL1, SNAIL2, TWIST1, and ZEB1 [52]—could improve our understanding of the systemic aspects of the disease. These genes show increased expression in the serum of women with endometriosis compared to controls [52]. However, it is important to recognize that blood-derived markers may not directly reflect the EMT processes specific to ectopic lesions. Future studies integrating both systemic and lesion-specific analyses may fill this gap and provide a more holistic view of EMT in endometriosis.

## 5. Conclusions

Our study highlights a potential connection between PNX-14 concentrations and cellular EMT transformations in endometriosis. Lower PNX-14 levels, typical in endometriosis patients, were associated with reduced *THBS2* expression, potentially driving increased transformation activity and inversely correlating with cell viability. In contrast, physiological PNX-14 levels may counteract these effects. These findings position PNX-14 as a promising therapeutic target. Elucidating its interplay with cellular transformation processes and estrogen within ectopic endometrium could advance treatment strategies for endometriosis.

## Figures and Tables

**Table 1:** Primer sequences for *B2M, CDH1,* and *THBS2* used in this study.

Gene	NCBI Accession No	Forward Primer (5′→3′)	Reverse Primer (5′→3′)	Probe No
*B2M*	NM_004048.4	GATGAGTATGCCTGCCGTGT	CTGCTTACATGTCTCGATCCCA	^1^
*CDH1*	NM_001270	CCACCAAAGTCACGCTGAA	TGCTTGGATTCCAGAAACG	#77
*THBS2*	NM_003247.2	CTTTGCGGAAAATGAAACG	TGATTTGGTGGCAAATGGT	#77

^1^ The locked nucleic acid probe #77 sequence (5′→3′): FAM-GGTGGTGG-BHQ.

## Data Availability

The raw data required to reproduce the above findings are available to download from https://doi.org/10.34808/71qf-pr48.
